# 

**Published:** 1866-05

**Authors:** 


CONTENTS FOR MAY.

ORIGINAL CONTRIBUTIONS.

The Use of the Thermometer in Clinical Medicine. By E. C. Seguin, M. D. (Illustrated.) .......................... p. 193
Clinical Lectures on Diseases of the Eye. By E. L. Holmes, M. D...... 202
The Pathology and Treatment of Cholera. By Henry M. Lyman, M. D. 207
Ligature of the Primitive Iliac Artery for Traumatic Aneurism. By Ralph N. Isham, M. D.......... 222

PROCEEDINGS OF MEDICAL SOCIETIES.

Chicago Medical Society.......... 226
DeWitt County Medical Society.......... 231

SELECTED ARTICLES.

*Surgery*—On Chronic Ulcers. By Frederick C. Skey, F. R. S., etc...... 232
*Miscellaneous*—On a New and Ready Method of Producing Local Anæsthesia. By Benj. W. Richardson, M. A., M. D., etc .......... 232
Antagonism of Morphia and Atropia.......... 237
*Medical News*.......... 238

EDITORIAL.

Items.......... 239

